# Self-Amplifying Replicon RNA Vaccine Delivery to Dendritic Cells by Synthetic Nanoparticles

**DOI:** 10.3390/vaccines2040735

**Published:** 2014-10-16

**Authors:** Kenneth C. McCullough, Panagiota Milona, Lisa Thomann-Harwood, Thomas Démoulins, Pavlos Englezou, Rolf Suter, Nicolas Ruggli

**Affiliations:** Institute of Virology and Immunology, CH-3147 Mittelhaeusern, Switzerland; E-Mails: panagiota.milona@ivi.admin.ch (P.M.); l.j.thomann@bluewin.ch (L.T.-H.); thomas.demoulins@ivi.admin.ch (T.D.); pavlos.englezou@ivi.admin.ch (P.E.); rolf.suter@gmx.net (R.S.); nicolas.ruggli@ivi.admin.ch (N.R.)

**Keywords:** self-replicating replicon RNA, targeting dendritic cells, nanoparticle delivery

## Abstract

Dendritic cells (DC) play essential roles determining efficacy of vaccine delivery with respect to immune defence development and regulation. This renders DCs important targets for vaccine delivery, particularly RNA vaccines. While delivery of interfering RNA oligonucleotides to the appropriate intracellular sites for RNA-interference has proven successful, the methodologies are identical for RNA vaccines, which require delivery to RNA translation sites. Delivery of mRNA has benefitted from application of cationic entities; these offer value following endocytosis of RNA, when cationic or amphipathic properties can promote endocytic vesicle membrane perturbation to facilitate cytosolic translocation. The present review presents how such advances are being applied to the delivery of a new form of RNA vaccine, replicons (RepRNA) carrying inserted foreign genes of interest encoding vaccine antigens. Approaches have been developed for delivery to DCs, leading to the translation of the RepRNA and encoded vaccine antigens both *in vitro* and *in vivo*. Potential mechanisms favouring efficient delivery leading to translation are discussed with respect to the DC endocytic machinery, showing the importance of cytosolic translocation from acidifying endocytic structures. The review relates the DC endocytic pathways to immune response induction, and the potential advantages for these self-replicating RNA vaccines in the near future.

## 1. Introduction

**Figure 1:**
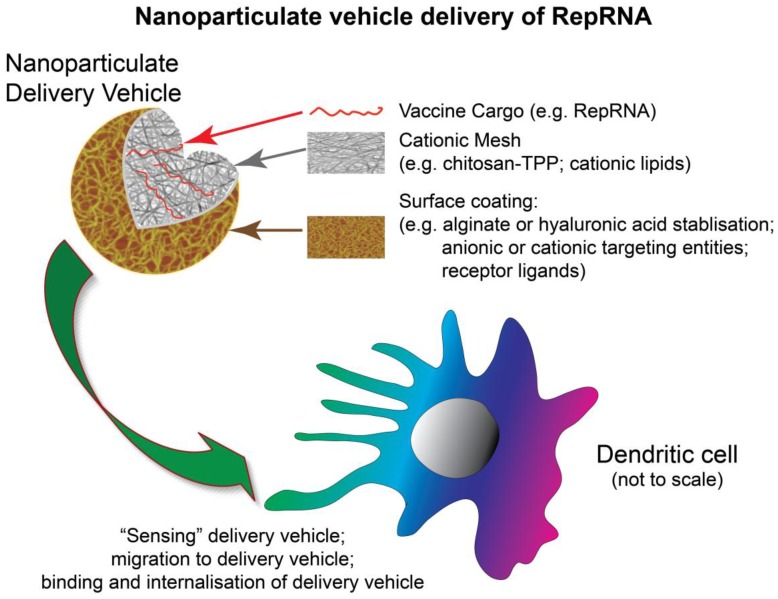
Delivery of RepRNA to DCs by nanoparticulate delivery vehicles. The delivery vehicles can be composed of polysaccharides, lipids, lipoproteins or combinations thereof. The nature of the delivery vehicle composition is to provide encapsulation of the RepRNA to protect against RNases, facilitate delivery to DCs and ensure a level of compaction enabling the RepRNA to interact with the ribosomal translation machinery. The surface of the nanoparticulate delivery vehicle may be coated to enhance stability and/or provide a means of enhance targeting of the DCs.

Dendritic Cells (DCs) play crucial roles in promoting and regulating immune responses, including adaptive immune defences. An important aspect is their capacity for handling antigens for delivery and presentation to the adaptive immune defence compartments [1]. Advances over the past two decades have expanded synthetic delivery of vaccine antigens, particularly when employing biocompatible, biodegradable nanoparticulate delivery vehicles (Figure 1). Nanoparticulate formulations have proven capacities for facilitating protein (drug or antigen) uptake by DCs. However, we recently demonstrated that the antigen cargo is an important component in terms of targeting cell receptors [2]. RNA does not possess this capacity. Moreover, delivery of antigen requires endosomal or protoeosomal processing, which would not guarantee survival of an RNA vaccine or its requisite delivery to the cellular translation machinery. Likewise, delivery of RNA for interference therapy (RNAi) cannot ensure efficient delivery of RNA for translation; the RNA for RNAi has particular requirements and intracellular targets distinct from those for RNA translation.

Although there are reports claiming targeting of DCs, many of these do not actually study interaction with the cells, presuming that an induced immune response reflected DC delivery. While this may be the case, it is not guaranteed for nucleic acid delivery. DNA vaccines can be delivered to promote immune response development, but the process of DNA nuclear translocation would not provide the efficient means of RNA delivery. An important property offered by nucleic acid vaccines relates to the initiation of potent immune responses benefitting from vaccines resembling the natural infection of the pathogen in question [3,4]. This becomes more difficult to achieve with vaccines that are inactivated and therefore non-replicating, although application of adjuvants can assist with vaccine efficacy [5]. An alternative approach is the application of RNA-based vaccines. As with vaccines employing inactivated antigen, the value of DCs and their diversity of receptors is most important, particularly for the required outcome of promoting translation of encoded vaccine antigens in the RNA.

The DCs therefore offer valuable targets for RNA vaccine delivery, being so central to promoting immune response development. Accordingly, this review will discuss how recent advances in RNA and biocompatible delivery vehicle technologies are advancing RNA vaccine development. The main aims focus on nanoparticle structures enhancing DC interaction, the consideration of cellular endocytic pathways for determining the outcome of vaccine delivery, and the application of self-amplifying replicon RNA (RepRNA) vaccines. As with mRNA vaccines, RepRNA vaccines require appropriate cytosolic delivery and transfer to the site of translation. Of particular importance are the roles of targeting ligands influencing delivery and intracellular compartmentalization, and cationic elements for cytosolic translocation. The high potential of the RepRNA delivery for vaccine applications will be elaborated in this review, proposing how progress will lead to enhanced self-amplifying vaccine targeting to DCs.

## 2. The Self-amplifying Replicon RNA

**Figure 2:**
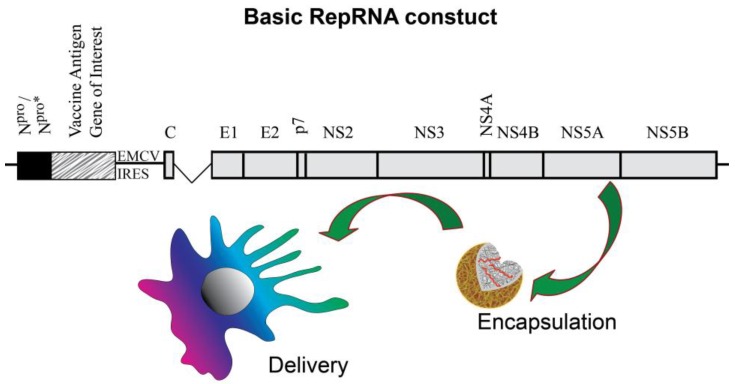
The basic RepRNA construct ensures efficient translation of the encoded vaccine antigen of interest, as well as replication of the replicon. Insertion of an internal ribosomal entry site (IRES) from, for example, EMC virus ensures that translation of the proteins for replication continues after translation of the vaccine antigen of interest.

RepRNA is derived from defective virus genomes (Figure 2). While RepRNA efficiently replicates and translates its encoded antigens, the defect prevents production of progeny virus [1,6,7,8,9,10]. This provides biosafe products, which in turn are readily engineered for carrying genes encoding vaccine antigens (Figure 2). A most important characteristic of RepRNA, offering a major advantage over inactivated or otherwise non-replicating vaccines, is the mimicking of virus replication in the sense of providing several rounds of replication; this increases mRNA templates and therefore enhanced antigen provision beyond the quantity possibly by conventional protein-based vaccines. This affords characteristics associated with efficient induction of both humoral immunity and cytotoxic cell-mediated immunity (CMI).

When RNA is delivered to DCs, it is necessary that it reach the translation machinery to translate its encoded antigens, as well as its polymerase proteins for replication of a RepRNA [1,6,7,8,9,10]. RNA molecules can also be retained in the maturing endosomal system, or re-introduced via autophagocytosis, in which case they can potentially interact with late endosome-like structures carrying Toll-like receptor (TLR) 3 or 7 [11,12,13,14,15,16]; the former TLR will detect dsRNA structures, which exist also with mRNA and RepRNA molecules due to the presence of hairpin loops [17], while TLR7 detects ssRNA motifs. Ligation of TLR3 or TLR7 by RNA molecules can lead to the induction of type I interferon (IFN) by DCs [11,12,13,14,15,16], wherein the plasmacytoid DCs (pDCs) are recognized as particularly potent producers of IFNα. Both pDCs and conventional DCs (cDCs) can also detect cytosolic RNA molecules (ssRNA and dsRNA) through their RIG-I-like sensors [12,15]. For ssRNA, this has to be distinguished from cellular RNA molecules, whereby it was shown that RIG-I sensing of ssRNA requires the presences of a 5'-triphosphate moiety. Both RIG-1 and MDA-5 can sense dsRNA molecules, whereby RIG-I binds stably with blunt-end, or 5' overhang molecules [12]. Interestingly, it has been reported that DC sensing of mRNA molecules more efficiently recognizes the dsRNA than ssRNA structures [17]. However, RNA molecules interacting with these cytosolic sensors are also unlikely to be available to the cellular translational machinery. Nevertheless, delivery of RNA vaccines to DCs has potential for delivering molecules to both translation and sensing compartments. Concerning RepRNA, that derived from the pestivirus classical swine fever virus (CSFV) carries a 5' autoprotease-encoding gene that possesses type I IFN regulatory activity [18,19,20]. When mutated to eliminate the IFN regulatory while retaining the autoprotease activity, both the virus and the replicon now induce type I IFN due to the dsRNA intermediates produced during replication [21,22]. Such IFN-inducing capacity did not appear to influence humoral responses induced by the RepRNA vaccine, but did enhance B- and T-lymphocyte recall responsiveness [22].

A major problem for RepRNA application has been its high RNase sensitivity, for which reason delivery has employed virus-like particle vectors: virus-like replicon particles (VRPs) [6,7,8,9,10,22]. This in turn has the drawback of requiring complementing cell lines to package the RepRNA into VRPs, thus necessitating expensive, specialised infrastructures. Moreover, VRP delivery is dependent on the cell tropism of the packaging particle, which is not readily modified for enhanced and controllable interaction with DCs. VRPs may also suffer from species or individual restriction, and questions exist concerning their stability over long periods. Application of VRPs can be bypassed by transfecting DCs *in vitro* prior to their transfer into vaccines [23]; unfortunately, these authors employed a DC cell line rather than primary DCs, so it is difficult to appreciate the validity of such an approach for primary DC targeting.

**Figure 3:**
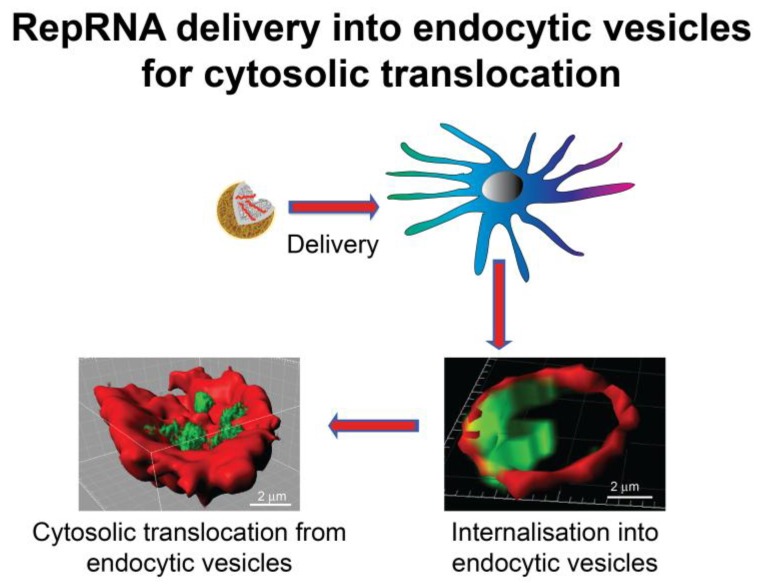
Nanoparticulate delivery of the RepRNA is designed to promote efficient uptake into endocytic vesicles, in which the RepRNA can be seen to accumulate. Thereafter, a gradual cytosolic translocation of the RepRNA from the endocytic vesicles, probably promoted by acidification of the vesicles, is essential to ensure delivery of the RepRNA to the intracellular site for RNA translation. It is considered that the acidification process, together with activation of the cellular redox processes facilitated destabilisation of the delivery vehicle and RNA, to permit entry of the ribosomes for translation.

An alternative to VRP-based delivery of RepRNA vaccines is application of biodegradable nanoparticulate vehicles (Figure 1), which have shown high potential for delivering to DC [1,24,25]. Replicon RNA has been delivered by coating on to gold microparticles [26], but these are neither biodegradable nor nanoparticles, and do not offer the advantages of biodegradable nanoparticles for targeting DCs and processing via DC endocytic pathways. While mRNA delivery to DCs has proven successful [27,28,29,30,31,32], it has only recently been shown that such nanoparticulate delivery is feasible for the much larger RepRNA molecules [33] (Figure 2 and Figure 3). This success of nanoparticle-based delivery of RepRNA to DCs requires an appreciation of the DC requirements for interaction with the delivery vehicles and the subsequent intracellular delivery of the RepRNA to the RNA translation sites (Figure 3).

## 3. Dendritic Cell Handling of Vaccines

Appropriate targeting of protein-based vaccines to DC can promote processing of the antigen through the DC endosomal system for presenting derived peptides in association with MHC Class II molecules to Th-lymphocytes [1,34,35,36,37,38]. DCs can also deliver more intact antigen to B-lymphocytes, as noted in the lymph node and spleen follicles [39,40]. This can be seen in terms of DC readily accumulating unprocessed antigen in macropinosomes and structures relating to CD63^+^ or Lamp1^+^ (CD107a) late endocytic compartments, for release in a small Rab27-dependent manner. Interestingly, similar late endosomal structures under Rab27 control are involved in processing for the MHC Class II presentation to Th-lymphocytes. This demonstrates that the DC endocytic pathways do not necessarily lead to protein antigen degradation. The cross-presentation pathways also employ routes involving endosomal escape, leading ultimately to the proteosome for processing and association with MHC Class I molecules in the endoplasmic reticulum (ER) [1,41,42,43,44,45]. In a similar sense, it is important that RNA vaccines do not undergo the processing leading the RNA into TLR3/7-containing compartments. Delivery must promote cytosolic translocation to the sites for RNA translation. Thereafter, the DCs can channel the translated antigen for processing and presentation to T-lymphocytes, as well as delivery to B-lymphocytes for their activation.

Efficient induction of lymphocyte function following vaccination requires vaccine interaction with the conventional DC (cDC) subset [46], referred to as the “professional antigen-presenting cell” (due to their distinctiveness as the most potent cells presenting antigen to naïve T lymphocytes and cross-presenting exogenous antigen and apoptotic cells). DC subsets can be classified dependent on their site in the body and activity in immune defence development [46]. CD103^+^ lamina propria DC apparently arise from the same lineage and pre-cDC as blood cDC; CX3CR1^+^ lamina propria DC arise from the same lineage (in the mouse) as monocytes and inflammatory macrophages; Langerhans cells may also arise from this linage. Regulatory DC subsets also exist, particularly at mucosal surfaces, or induced by their environment. Targeting the appropriate DC subsets is crucial for efficient vaccine delivery leading to adaptive immune response development. While the receptors displayed on DC subsets have a strong influence, this is not merely in terms of targeting vaccine interaction with the appropriate subset, but also the consequence of that interaction in terms of the endocytic pathways employed by the DCs.

## 4. Endocytic Processing Pathways of Dendritic Cells

Vaccine delivery to DCs will encounter the high capacity of DCs for internalizing a wide variety of antigen forms and types, through their diverse network of endocytic routes [1,47,48,49,50,51]. These can be classified in terms of their dependency or independency on clathrin, caveolin, lipid rafts and dynamin. Nanoparticle delivery of vaccine to DC must therefore consider this variety of DC endocytic processes. The route of endocytosis will depend on the material being delivered, and the mechanism of delivery to the DCs. This dependency or not on clathrin, caveolin and dynamin has given rise to the terms clathrin-dependent endocytosis, caveolar endocytosis, and macropinocytosis. Phagocytosis is often employed to describe endocytosis of large bodies, contrasting with macropinocytosis, which has been related more to fluid-phase uptake, although this description is far from being definitive or absolute. Endoplasmic reticulum (ER)-phagocytosis refers to elongation of tubule-like structures from the ER, interacting with phagosomal structures. Regardless of the route, the endocytosed material must be channelled into the appropriate structures for antigen processing or RNA translation, to promote ultimately antigen delivery to B-lymphocytes or processing for presenting to T-lymphocytes.

There are certain crucial requirements involved in efficient processing of antigen, which also have a major bearing on RNA delivery and the likelihood of its translation. The endocytic processing pathways have been widely studied in the context of antigen presentation to Th lymphocytes [36,37,38]. The endocytosed material must encounter endosomes, which provide the vacuolar H^+^-ATPases necessary for gradual acidification of the endocytic vesicle in which the antigen is found, as well as the proteases (carboxypeptidases, aminopeptidases, and endoproteases) necessary for the processing. These proteases (cathepsins) are pH-sensitive; hence the gradual acidification to pH 5.5 as the endosome matures from an “early endosome” to a “late endosome”. An important consequence therein for RNA delivery is the escape of the RNA from this acidifying system prior to attack by the RNases. In this context, the action of the vacuolar H^+^-ATPases plays an important role, in a similar fashion to its role in initiation of the cross presentation pathways for antigen.

## 5. Learning from Cross-Presentation Pathways in Dendritic Cells

As with DC processing of antigen for presentation to Th lymphocytes, both exogenous and endogenous antigen can be processed for Tc lymphocytes. This processing of exogenous material via “cross-presentation” pathways [34,35,43,44,45] is important for activating the Tc lymphocytes of cytotoxic CMI. Characteristics of these pathways may prove pertinent to RNA translocation from the endosomal system. As with MHC Class II processing, cross presentation can utilize different endocytic processes employed by DC [34,44], but may involve particular DC subsets. Again, the endocytic route will be dependent on the manner of targeting and the receptors involved. The receptors binding the delivery vehicle prior to endocytosis will influence the form of endocytosis, in particular macropinocytosis and caveolar uptake, which can also deliver the internalized material into the ER [44]. While targeting delivering into early compartments results in both MHC Class I and Class II presentation, delivery into late endocytic compartments leads to domination of MHC Class II presentation [42,52]. Delivered material for MHC Class I presentation may be in similar endocytic or autophagocytic vesicles as material destined for MHC Class II presentation. In contrast, the processing of antigen for association with MHC Class I molecules can be regarded as a less acidic process, or even a neutral pH process. For example, delivery of ER membranes to phagosomes for insertion of the ER dislocon leads to antigen associated with ER-like phagosomes, which can be cross-presented [41,44].

An important issue pertinent to nanoparticle delivery is the size of the material endocytosed; this influences both the endocytic route of uptake and the consequences on cell handling of the endocytosed material [35,44]. Smaller, more “soluble” material may be transferred from endocytic vesicles into the ER by retrograde transport [35], an important consideration with RNA translation requiring delivery to cytosolic sites associated with the ER. Macropinocytosis and caveolar endocytic delivery to the ER may occur without interaction with early endosomes, or shortly after acidification begins. Importantly for RNA delivery, the macropinosomes and caveolar vesicles may deliver their contents to the cytosol.

It would appear that the majority of endocytosed material reaching macropinosomes accumulates in lysosomes [1,44,49,50,51], which would prove problematic for RNA. Yet, the relatively low degradation rate following macropinocytosis would facilitate retrograde transport into the ER [41,42,44,45,50]. In a similar vein, caveolar endocytosis, which can also provide lower acidification and degradation rates, facilitates vesicular transport to the ER, as witnessed with simian virus 40 [48,53,54] A limiting feature is the rate of acidification and augmentation of endosomal enzyme activity [1,42,48,53,54]. It is necessary to consider that RNA delivery to DCs may lead to a majority entering the potentially more destructive later endosomal structures, with a minority translocating earlier to the cytosol and/or regions rich in ER. Amigorena and Savina [42] elaborated on this by comparing macrophages with DCs in their review on how common endocytic process are used for both MHC Class I and Class II pathways. Macrophages rapidly degrade endocytosed material due to rapid recruitment and activation of lysosomal proteases. In contrast, DCs more slowly degrade internalized proteins, and their endosomal pH can be less acidic. DCs also express activated NOX2 subunit of NADPH-oxidase. This generates reactive oxygen species in endocytic compartments, which consume protons and therefore modulate the acidifying pH. Both the effect on vesicular pH and the generation of reactive oxygen species have important consequences for RNA delivery, in terms of cytosolic translocation and release of the RNA from the delivery vehicle. A more prolonged maintenance of higher pH in the early endocytic compartments of DCs compared with macrophages may facilitate the cytosolic egression of antigen seen with cross-presentation, and may prove an ally in assisting RNA cytosolic translocation. Indeed, cytosolic translocation does not always require the involvement of the ER. Hamdy *et al*. [24] proposed that nanoparticle-delivered antigen might escape from the endosomal system to be slowly hydrolysed in the cytosol for release of antigen to be processed by the proteosome. This may prove a potential route for release of RNA to promote translation.

## 6. Targeting DC with RNA Vaccines

Many vaccines in current use are non-replicating entities. Although nanoparticle delivery vehicles have been widely applied in vaccinology, a major focus has been humoral immunity; indeed, the capacity for promoting the various arms of immune defence may prove limited. Replicating vaccines by their nature may provide conditions more related to when immune defences develop following pathogen infection; thus, a greater capacity for inducing both humoral and cellular (cytotoxic) immune defences is conceivable. Such vaccines cannot be created for all pathogens, and live vaccines present a risk of reversion to more pathogenic progeny.

While application of DNA vaccines has received much attention, the DC nuclear membrane can prove quite resistant to nucleic acid translocation [55,56]. This problem does not arise with RNA vaccines, which must target cytosolic sites of translation. They also do not present the biosafety risks potentially associated with DNA vaccines [56]. A major focus for nanoparticulate vehicle-based RNA delivery has not been with vaccines but on delivering small interfering RNA (RNA), small hairpin RNA (shRNA) or double-stranded RNA (dsRNA) for RNA interference (RNAi) therapy [57,58,59,60,61,62,63,64]. This approach is not directly transferable to RNA vaccine delivery, due to the different intracellular compartments to which RNA vaccines need to be delivered compared with RNA for RNAi [1,61,63]. When RNA vaccines are targeted to DC (Figure 3), this must lead to translation of the RNA to provide the antigenic components for promoting adaptive immunity. Unlike protein-based, lipid-based and carbohydrate-based antigens, RNA binding to DC receptors is inefficient or even impossible to demonstrate. This is where nanoparticulate delivery vehicles come into their own, not only for enhancing delivery to DCs (Figure 3), but also protecting the RNA from RNases.

## 7. DC Vesicular Acidification and Cytosolic Translocation

While many studies on DC endocytosis have focused on clathrin-mediated uptake [65], the rapid internalization and acidification associated with this process may lead more to degradation or delivery into TLR-containing endosomal structures. For uptake leading to translation, one must consider macropinocytosis, caveolin-mediated endocytosis and lipid raft-dependent processes [66]. An important factor regardless of the endocytic route is interaction with early endosomes and ultimate acidification due to the action of vacuolar H^+^-ATPases. A major difference among the different routes is the rate at which this occurs, clathrin-mediated endocytosis tending to appear the more rapid [34,47,48,49,50,51]. Additional structures such as sorting endosomes can also be involved in determining the prolonged accumulation of endocytosed material before involvement with acidifying endosomes. In contrast to macrophages, DC tend to show more gradual processing following internalization, and retention of the material for longer periods.

An important consequence for successful RNA vaccine delivery must be cytosolic translocation from the endocytic vesicle to permit interaction with the cellular translation machinery. Such cytosolic delivery can be enhanced by modifying the consequences of endosomal acidification on the endocytic vesicle integrity [67,68]. When cationic delivery vehicles such as cationic liposomes are employed, they efficiently encapsulate nucleic acids, to form polyplexes and lipoplexes, and deliver RNA for translation [67,69,70]. Chitosan-based nanogels have also been employed in this sense [33,71].

It is considered that structures such as chitosan and PEI containing protonable amines (Figure 1) can buffer due to these groups accepting protonation. When vacuolar H^+^-ATPases pump protons into the acidifying endocytic vesicle, such nanoparticulate structures can provide the so-called “proton sponge effect” [71]. Certainly, amino and cationic groups are important for protonation facilitating cytosolic translocation from endocytic vesicles [72]; histidine-rich and arginine-rich molecules, or histidine residues as polar heads, can also initiate a proton sponge effect through protonation of imidazole rings [67,68]. The consequence of protonation increases ion and water uptake and thus osmotic pressure within the vesicles. Subsequent vesicular swelling leads to membrane disruption, the consequence of which is cytosolic release.

The proton sponge effect is not the only manner by which cytosolic translocation may be promoted. Interaction with the anionic vesicular membrane is an important consideration [68], potentially reducing internal membrane tension when binding at the edges of membrane pores. Amphiphilic entities can insert into the vesicular membrane, promoting internal membrane tension as acidification of the endocytic vesicle progresses; again membrane rupture for cytosolic release can ensue [67,73]. Peptides that can perturb vesicular membrane integrity through structural modifications, including fusiogenic activity, have also been employed for cytosolic translocation of nucleic acids [68,73]. Combining the properties of more than one approach may also prove beneficial [65], as can the employment of “helper” lipids such as 1,2-dioleoylphosphatidylethanolamine (DOPE) and cholesterol [74].

## 8. Learning from Cytosolic Translocation of Small RNA Molecules

Although delivery of oligonucleotides for RNA interference therapy have successfully employed cationic-based delivery vehicles [1,58,59,61,67,75], siRNA and miRNA are much smaller molecules than mRNA or RepRNA. Moreover, they interact with particular structures in the cell for promoting interaction with mRNA sequences leading to destruction or inhibition of the mRNA [76]. Delivery of RepRNA can learn more from mRNA delivery, due to both molecules requiring interaction with the cellular translation machinery. The work on mRNA delivery has shown the value of nanoparticulate delivery vehicles such as mannosylated/histidylated lipopolyplexes [31,77,78], cationic liposomes [79], or copolymer blends of PEI and PEI-PEG and cationic lipids 1,2-dioleoyl-3-trimethylammonium propane (DOTAP) and DOPE [80,81] (Figure 1). Nevertheless, mRNA is again a much smaller molecule than RepRNA. As with many of the DNA vaccines, which are also smaller molecules than the RepRNA, a major influence and therefore consideration is the different N:P ratio when associating nucleic acids of different size with the delivery vehicle. Interaction of the nanoparticle amines with the RNA phosphates is critical for ensuring efficient encapsulation and therefore delivery of protected (from RNases) RNA. On the other hand, the interaction must not be too strong to result in compaction, preventing sufficient RNA vaccine dissociation to facilitate efficient interaction with the ribosomes. Elucidation of the conditions required for efficient delivery allowing intracellular dissociation for efficient interaction with ribosomes is still an ongoing study. Nevertheless, it is now clear that nanoparticulate vehicles can deliver both mRNA and RepRNA to DCs (Figure 3), in a manner promoting translation of the RNA molecules.

## 9. RepRNA Delivery to Dendritic Cells for Encoded Antigen Translation

With self-amplifying RepRNA offering high potential in vaccinology due to its replicative nature [1], critical issues remain concerning the mode of delivery and interaction with DCs. Due to the problems associated with the more widely employed use of VRP delivery [6,7,8,9,10,22]—requirement for complementing cells to supply the deleted gene and thus allow VRP formation, potential species restriction, anti-VRP immunity, targeting cells other than DCs—nanoparticulate delivery vehicles offer a potentially successful alternative. It is also important to consider the nature of the RepRNA, which will relate back to the parent virus from which it as derived. The widely studied cytopathogenic alphavirus replicon vaccines [6,7,8,10] rapidly kill host cells. This in turn would pose problems for DCs, considering their characteristics of slow processing and retention of antigen for prolonged interaction with the adaptive immune system. Non-cytopathogenic replicons from flaviviruses [7,9] and pestiviruses such as CSFV [22] (Figure 2) are more in line with such DC characteristics.

The potential for biocompatible nanoparticulate vehicle delivery of RepRNA to DCs was proposed as early as 2008 [82] (Figure 2); following the presentation of this work, the concept that nanoparticles could be employed for RepRNA delivery was confirmed *in vivo* [83]. However, the latter report used a modified RNA molecule that could be employed without any delivery vehicle. We have demonstrated that unmodified RepRNA (not capped, not carrying a poly(A) tail, nor modified with protein for protection against RNase and promoting delivery) (Figure 2) could be successfully delivered to DCs (Figure 3) and induce immune responses *in vivo* [33]. RepRNA derived from the non-cytopathogenic classical swine fever virus (CSFV) (Figure 2) was efficiently associated with chitosan-based nanoparticles coated with alginate (Figure 1), referred to as nanogels (NGA) due to their matrix-like formation. The RNA cargoes were seen to be delivered into vesicular structures within DCs (Figure 3), from which cytosolic translocation of the RepRNA seemed evident, considered to be reflecting reduced pH in the vesicle increasing amine protonation on chitosan [84] for destabilizing the acidifying endocytic vesicles [1,32,68,74,85]. Incorporating cationic lipids with the chitosan during NGA formulation appeared to enhance the efficiency of delivery RNA into DC vesicular structures, particularly notable in terms of the RNA translation efficiency. Moreover, nanoparticle delivery of RepRNA was reported to be successful for both mice and rabbits [33], which has also been recently confirmed [86].

This article also confirmed that differences could be observed for RepRNA and oligoRNA delivery [33]. Not only was there a distinction in terms of the cell subsets targeted, there were also differences in the rates of internalization and intracellular accumulation. The authors proposed that this might relate to differences in recycling of oligoRNA and RepRNA by the cell subsets; this in turn would reflect the different sites to be targeted for successful RepRNA delivery compared with oligoRNA such as molecules for RNAi. For the RepRNA, NGA delivery led to RNA accumulation in vesicular structures typical of endocytic elements such as macropinosomes [1,48,49,51,87] (Figure 3). With time, weaker RepRNA signals emerged adjacent to these vesicular structures, which the authors suggested were indicative of cytosolic translocation based on reports for nanoparticle delivery of oligoRNA to Hela cells [88,89]. Regardless of such image characteristics, the important outcome for successful RepRNA delivery has to be the translation of the RNA. Translation of an encoded luciferase, as well as the RepRNA endogenous NS3 gene were found in DCs. Expression of the latter was an important first step to replication of the RepRNA, which is essential for the RepRNA to display its self-amplifying characteristics. Moreover, replication would enhance the likelihood of the DCs facilitating induction of both humoral immunity and CMI. Replication was confirmed in terms of the translation kinetics being maintained at high levels over a number of days, something which is not possible when the RepRNA carries a mutation in genes of its polymerase complex [90].

## 10. RepRNA Delivery by Nanoparticulate Vehicles Induces Immune Responses *in Vivo*

The observed successful delivery of pestivirus RepRNA to DCs provided the necessary RepRNA translation *in vivo* for inducing immune responses, observed in both mice and rabbits [33]. When the analyses were extended to monitor both humoral immunity and CMI, antigen-specific antibody as well as both T_h_-lymphocyte and T_c_-lymphocyte immunity were identified. These responses were monitored with reference to anti-HA and anti-NP activity induced by the influenza virus HA and NP encoded by the RepRNA. Interestingly the authors employed two RepRNA molecules, each encoding one of the influenza virus antigens, and each encapsulated individually. Vaccination employed an equimolar mixture of the two formulated RepRNA, which proved to be a clearly successful approach. An additional report using an alphavirus replicon with *in vivo* liposome-like delivery [83] was also apparently successful, although there was no analysis of DC interaction; indeed the observation that replicon translation occurred at the site of inoculation argues against interaction with DCs. Moreover, the authors reported that their “naked” RNase-sensitive replicon was also effective at inducing immune responses *in vivo*. Such an observation cannot be repeated with an RNase-sensitive pestivirus RepRNA, which does not lead to translation or induction of immune responses *in vitro* or *in vivo* when employed as “naked” RNA [33]. These observations may relate to the application of mRNA vaccines applied as “naked” RNA; such vaccines require capping and/or poly-adenylation [1,28,91,92,93]. It has been well established for over two decades that alphavirus RNAs contain 5' hypermethylated caps, together with a 3' poly(A) tail in their genomic structure [94]. The 5' cap is essential for alphavirus RNA translation [95,96] and therefore genomic replication (reviewed by Strauss and Strauss [94]), although it is still uncertain what role the poly(A) tail is playing (it is considered that this may be involved in minus strand RNA synthesis). Accordingly, it is also necessary to cap the 5' of the alphavirus replicon [97,98]; Rossi *et al*. [98] stated that the 5' cap was required for replicon RNA replication. Current alphavirus replicon vaccines are capped using, for example, the Vaccinia Capping system [83]. It would appear that such capped replicon molecules can be employed without the need for a delivery vehicle [83,86], related to what has been reported for mRNA vaccines [1,28,91,92,93]. Nevertheless, not all RNA viruses require capping or poly(A) tails. Some RNA viruses initiate translation in a cap-independent manner via internal ribosomal entry, as exemplified by the pestivirus CSFV [99]. Such viruses employ an internal ribosomal entry site (IRES) in the 5'-NTR to initiate translation [99,100]. Thus, RepRNA derived from viruses such as CSFV also follow cap-independent translation initiation, which can offer advantages in not necessitating manipulation of the replicon as in the case of alphavirus replicon capping using the Vaccinia Capping system. Nanoparticulate delivery of such a cap-independent RepRNA can be successful, for both delivery to DCs and initiation of immune response induction [33].

It has also been reported that successful RNA delivery *in vivo* is achievable by complexing with BSA [101]. Employing replicons complexed with proteins or even VRPs, it is likely that nanoparticulate delivery vehicles would further assist their delivery. Under such conditions, the additional proteins or VRP components would not necessarily be “hidden” by the delivery vehicle, as recently reported for ovalbumin delivery by chitosan particles [2]. Virions and VRPs interact with cell receptors, which lipid- or polysaccharide-nanoparticles cannot provide. Virions and VRPs also promote cytosolic translocation of the genome by rearrangement of virion capsid proteins to form cytosolic delivery channels or disruption of the endocytic vesicular membrane [54]; again not present with lipid- or polysaccharide-nanoparticles. It would be anticipated that nanoparticulate vehicle delivery of RepRNA complexed with proteins or VRP components capable of influencing interaction with cell receptors could provide a delivery efficiency similar to the use of VRPs, which would in turn be enhanced by manipulating the conditions of the delivery vehicle in terms of vehicle:cargo ratios.

The recent work on NGA-delivery of cap-independent RepRNA to DCs (Figure 2 and Figure 3) has demonstrated the high potential for the use of such RepRNA vaccines delivered by biodegradable, biocompatible nanoparticulate delivery vehicles [33]. Not only can the RepRNA be targeted to the DCs, its delivery can be manipulated to promote the cytosolic translocation necessary to ensure translation of the vaccine antigens encoded by the RepRNA. Moreover, the evidence points to the replication of this delivered RepRNA, which in turn would increase the number of mRNA templates available for translation and therefore the quantity of antigen produced. With this antigen being present in the DCs, both humoral immunity and CMI can be promoted, due to the inherent characteristics of the DCs for handling endogenous antigen. Of course, this would require a RepRNA that was not cytopathic for the DCs, to permit the cells to perform appropriately and efficiently; the use of cap-independent replicons (Figure 2) also offers advantages facilitating their production. The reported *in vivo* work [33] confirms that such RepRNA will induce both arms of the immune defence when delivered by nanoparticulate vehicles capable of interacting efficiently with the DCs.

## 11. Conclusions

To date, synthetic particle delivery of RNA has focused on siRNA and mRNA. There were no reports on RepRNA interaction with DCs until recently [33]. This work characterized chitosan-based nanoparticulate vehicle delivery (Figure 1) of self-amplifying, cap-independent replicon RNA (Figure 2) to DCs (Figure 3), promoting RepRNA translation and induction of immune responses. The RNase-sensitive replicon can be protected, eliminating a need for 5' capping or 3' poly(A) tail, and overcome the inability of “naked” replicon to function *in vivo*. For both humoral immunity and CMI, the inherent characteristics of the DCs for handling antigen can be employed by targeting the RepRNA vaccine to the cell type ensuring efficient immune defence induction. In this context, an important consideration is the application of a RepRNA non-cytopathic for the DCs. One aspect of this delivery to DCs that can be enhanced is the targeting element. Current work is pursuing this aspect to determine how the characteristics of targeting relate to particular endocytic processing and ultimately how this impacts on the efficiency of the RepRNA translation and replicon, leading to induction of both the humoral and cytotoxic arms of immune defense.
